# Chemical Diversity and Biological Activities of Meroterpenoids from Marine Derived-Fungi: A Comprehensive Update

**DOI:** 10.3390/md18060317

**Published:** 2020-06-15

**Authors:** Amr El-Demerdash, Decha Kumla, Anake Kijjoa

**Affiliations:** 1 The John Innes Centre, Department of Metabolic Biology, Norwich Research Park, Norwich NR4 7UH, UK; eldemerdash555@gmail.com; 2 Chemistry Department, Faculty of Science, Mansoura University, Mansoura 35516, Egypt; 3 ICBAS-Instituto de Ciências Biomédicas Abel Salazar & CIIMAR, Universidade do Porto, Rua de Jorge Viterbo Ferreira 228, 4050-313 Porto, Portugal; Decha1987@hotmail.com

**Keywords:** meroterpenoids, marine-derived fungi, biological activities, antibacterial, cytotoxicity, anti-inflammatory

## Abstract

Meroterpenoids are a class of hybrid natural products, partially derived from a mixed terpenoid pathway. They possess remarkable structural features and relevant biological and pharmacological activities. Marine-derived fungi are a rich source of meroterpenoids featuring structural diversity varying from simple to complex molecular architectures. A combination of a structural variability and their myriad of bioactivities makes meroterpenoids an interesting class of naturally occurring compounds for chemical and pharmacological investigation. In this review, a comprehensive literature survey covering the period of 2009–2019, with 86 references, is presented focusing on chemistry and biological activities of various classes of meroterpenoids isolated from fungi obtained from different marine hosts and environments.

## 1. Introduction

Meroterpenoids are a large group of secondary metabolites of mixed biosynthetic origin, partially derived from mevalonate pathways. Another part of these metabolites can be derived from other biosynthetic pathways, most of which are polyketides and, to a lesser extent, nonpolyketides such as amino acids [1]. Meroterpenoids are widespread in nature, being isolated from terrestrial plants [2], marine invertebrates [3], and microorganisms such as fungi [4] and bacteria [5,6]. Fungi not only are the most prolific producers of meroterpenoids but also synthesize structurally diverse metabolites of this group with a wide range of biological and pharmacological activities [4]. Consistently, Geris and Simpson [4] published the first review of meroterpenoids produced by fungi in 2009, covering the period of 1968 to August 2008. This review provided information on isolation, structure elucidation and some biological activities, in addition to a detailed discussion of biosynthetic studies of 333 fungal meroterpenoids. However, in most cases, there was no indication if the fungi under study were from terrestrial or marine origin. In 2016, Matsuda and Abe published a comprehensive review of the biosynthesis of fungal meroterpenoids, updating the biosynthetic information previously discussed in the review by Geris and Simpson by summarizing the molecular basis, elucidated by modern techniques, of various classes of meroterpenoids [1]. On the other hand, it is interesting to note that despite the discovery of cephalosporins from the marine-derived fungus *Cephalosporium acremonium* (which is known today as *Acremonium chrysogenum*) in 1948 [7], the interest in the investigation of secondary metabolites from marine-derived fungi only started in the 90s, with only 15 marine fungal metabolites reported by 1992 [8]. However, with the renewed interest in fungal biodiversity of the marine environment, the number of the isolated compounds kept rising to 270 in 2002 [9], and ramped up to 690 during the period of 2006 to mid-2010 [10]. From the literature search, it is evident that meroterpenoids constitute an important class of structurally unique secondary metabolites with relevant biological and pharmacological activities produced by fungi from nearly every possible marine habitat including soil and sediments, marine invertebrates (e.g., sponges, corals, sea cucumbers), marine plants (e.g., algae, sea glass, mangroves), and marine vertebrates (fishes) [11]. Moreover, they also display a myriad of biological activities including antioxidant [12], cytotoxic [13,14,15], antimicrobial [16,17], antiviral [18,19], anti-inflammatory [20], and anti-Alzheimer [21]. Despite this extraordinary increase in the research on natural products from marine-derived fungi, there is no systematic review of meroterpenoids from marine-derived fungi to date. Therefore, this review focuses on the chemistry and relevant biological activities of 320 meroterpenoids from marine-derived fungi reported in the literature over the period of 2009 to December 2019. Contrary to the classification based on the types of polyketides adopted by Geris and Simpson [4], herein we grouped the reported meroterpenoids according to the terpenoid classes, i.e., hemiterpenes, monoterpenes, sesquiterpenes and diterpenes. In this review, the biosynthesis aspects of this class of compounds are not discussed as they have been extensively reviewed by Geris and Simpson [4] and then updated by Matsuda and Abe [1].

## 2. Chemistry and Biology of Meroterpenoids Isolated from Marine-Derived Fungi

In this section, a comprehensive summary of 320 structurally diverse meroterpenoids isolated from the culture extracts of marine-derived fungi over the period of January 2009 to December 2019 is presented. All the isolated compounds were classified according to their featured terpenoid part, i.e., hemiterpenes, monoterpenes, sesquiterpenes and diterpenes. The relevant biological and pharmacological activities of the reported compounds are provided wherever applicable.

### 2.1. Merohemiterpenoids

#### 2.1.1. Merohemiterpenoids Containing Acyclic Hemiterpenes (Figure 1)

**Figure 1:**
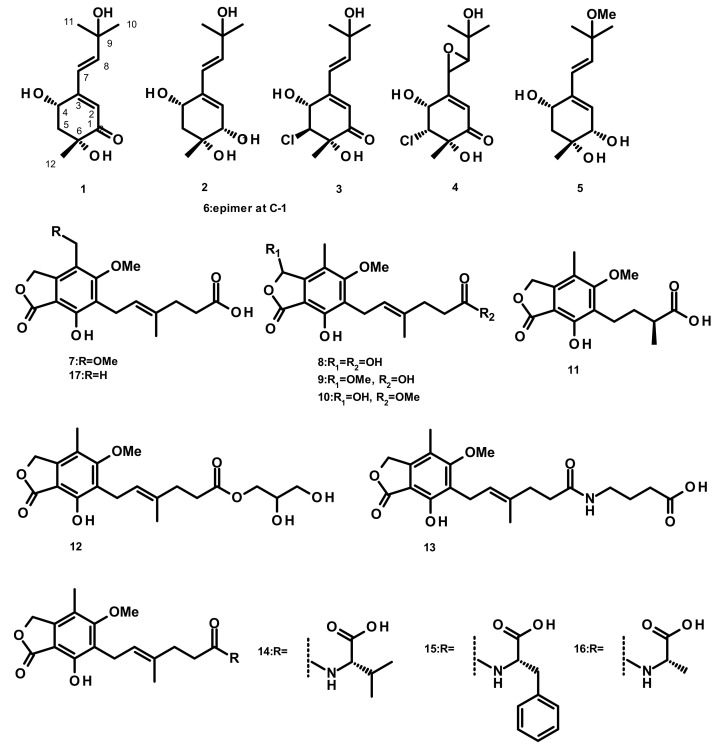
Chemical structures of acyclic merohemiterpenes **1**–**17**.

Six merohemiterpenes (Figure 1), acremine A (**1**), acremine F (**2**), 5-choloroacremine A (**3**), 5-choloroacremine H (**4**), 9-*O*-methylacremine F (**5**) and 1-*epi*-acremine F (**6**), were obtained from the culture of the marine-derived fungus *Acremonium persicinum*, which was isolated from the marine sponge *Anomoianthella rubra*. None of the isolated compounds were assayed for any biological activity [22]. Mycophenolic-acid-based merohemiterpenes **7**–**17** (Figure 1) were isolated from *Penicillium bialowiezense,* which was obtained from the soft coral *Sarcophyton subviride.* Compounds **7**–**17** exhibited an inhibitory activity against inosine-50-monophosphate dehydrogenase (IMPDH2) with IC_50_ values ranging from 0.59 to 24.68 µM. These compounds were also assayed for the in vitro immunosuppressive activity against the proliferation of T-lymphocytes, and **7**–**9** exhibited IC_50_ values ranging from 0.84 to 0.95 µM, whereas the IC_50_ values of **10**–**17** were from 3.27 to 24.68 µM [23].

#### 2.1.2. Merohemiterpenes Containing Cyclic Hemiterpenes (Figure 2)

**Figure 2:**
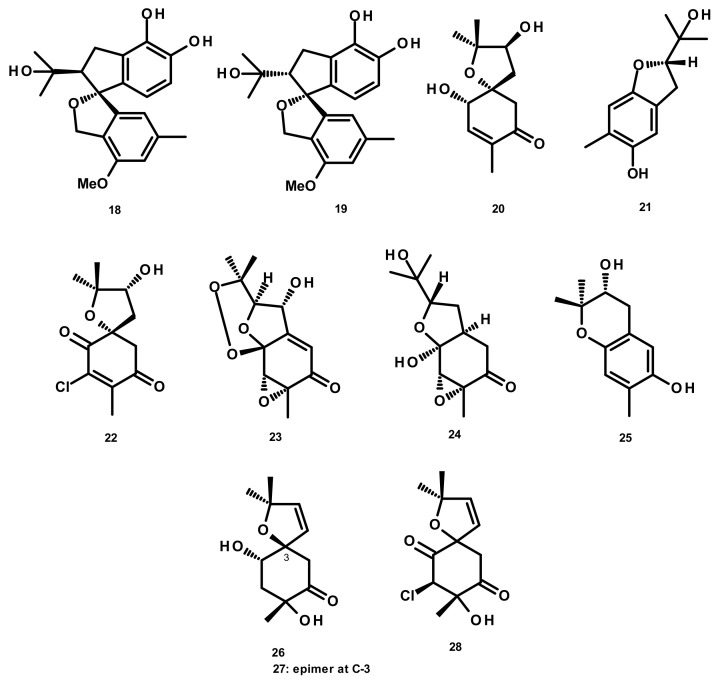
Chemical structures of cyclic hemiterpenes **18**–**28**.

Spiroarthrinols A (**18**) and B (**19**) (Figure 2) were isolated from the sponge-derived fungus *Arthrinium* sp., obtained from the inner tissues of a marine sponge *Sarcotragus muscarum* collected off the coast of Southern Turkey. Both compounds did not show any significant in vitro cytotoxic activity against the Caco-2 (human epithelial colorectal adenocarcinoma) cell line [24]. A bicyclic merohemiterpene, acremine S (**20**) (Figure 2), was recently isolated from the marine-derived fungus *Acremonium persicinum* KUFA 1007 which was isolated from the marine sponge *Mycale* sp., collected from the coral reef in the Gulf of Thailand. Although **20** exhibited a weak inhibitory activity against acetylcholinesterase (AChE), its activity against butyrylcholinesterase (BuChE) was threefold higher than that of the positive control galantamine [25]. Acremines N (**21**), O (**22**), P (**23**) Q (**24**), R (**25**), spiroacremines A (**26**) and B (**27**) and 5-chlorospiroacremine (**28**) (Figure 1) were isolated from the marine-derived fungus *Acremonium persicinum*, obtained from a marine sponge *Anomoianthella rubra*. However, no bioactivity of the isolated compounds was investigated [22].

### 2.2. Meromonoterpenoids (*Figure 3*)

**Figure 3:**
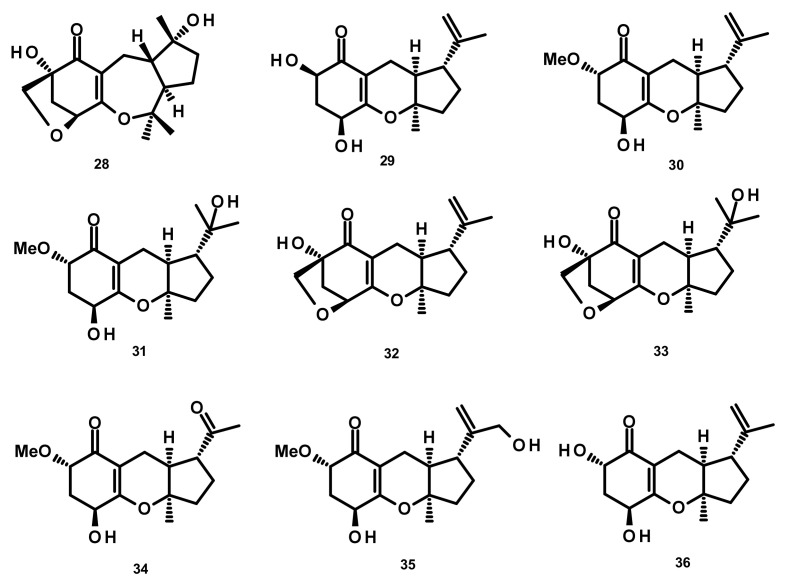
Chemical structures of meromonoterpenoids **29**–**37**.

Chemical investigation of the endophytic fungus A1, isolated from leaves of the mangrove plant *Scyphiphora hydrophyllacea*, afforded the previously unreported guignardones F–I (**29**–**32**), together with the previously described guignardones A (**33**) and B (**34**) (Figure 3). Compound **32** displayed antibacterial activity against methicillin-resistant *Staphylococcus aureus* (MRSA) and *S. aureus* with inhibition zones of 9.0 and 11.0 mm, respectively, whereas **34** exhibited inhibition activity against MRSA with a minimum inhibitory concentration (MIC) value of 65 µM [26]. The previously unreported guignardones J (**35**), K (**36**), M (**37**) (Figure 3), along with **31**, **33** and **34,** were obtained from the culture extract of the endophytic fungus *Aspergillus flavipes* AIL8, which was isolated from the inner leaves of the mangrove plant *Acanthus ilicifolius*. The isolated compounds showed neither antibacterial nor cytotoxic activities [27].

### 2.3. Merosesquiterpenoids

#### 2.3.1. Merosesquiterpenenoids Containing Acyclic Sesquiterpenes (Figures 4 and 5)

**Figure 4:**
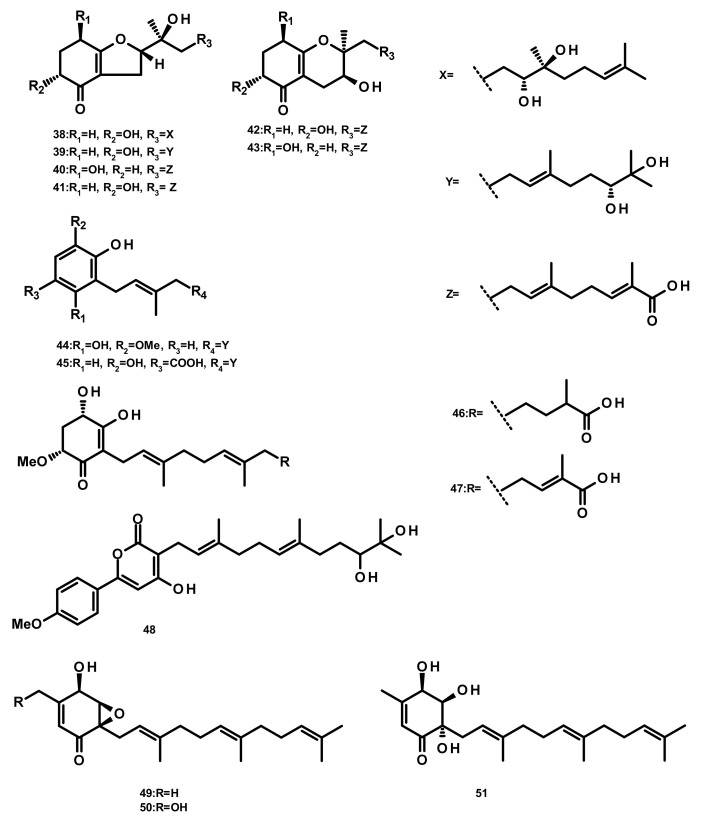
Chemical structures of acyclic sesquiterpenes **38**–**51**.

Merosesquiterpenes **38**–**47** (Figure 4) were isolated from the marine-derived fungus *Alternaria* sp. JJY-32, which was obtained from the marine sponge *Callyspongia* sp. Supplementation experiments with specific enzyme inhibitors and putative precursors led to the conclusion that they are originated from a shikimate-isoprenoid hybrid biosynthetic pathway. All the compounds, except **44**, displayed NF-κB inhibitory activities with IC_50_ values ranging from 39 to 85 μM in RAW264.7 cells [28]. Another merosesquiterpene named arisugacin I (**48**) (Figure 4), was obtained from the endophytic fungus *Penicillium* sp. SXH-65, which was isolated from the leave of *Tamarix chinensis* growing on a saline-alkaline soil on the coast of Laizhou Bay in Dongying, China. Compound **48** showed no cytotoxicity against HL-60 (human leukemia), K562 (chronic myelogenous leukemia) and Hela (cervix carcinoma) cells [29]. 7-Deacetoxyyanuthone A (**49**), 2,3-hydrodeacetoxyyanuthone A (**50**), 22-deacetylyanuthone A (**51**) (Figure 4), three merosesquiterpenes featuring a quinone/hydroquinone scaffold, were isolated from the marine-derived fungus *Gliomastix* sp. ZSDS1-F7, obtained from the marine sponge *Phakellia fusca* Thiele, which was collected on the Yongxing island of Xisha. Compound **50** displayed significant in vitro cytotoxicity against several cancer cell lines including K562, MCF-7 (breast cancer), Hela, DU145 (prostate cancer), U937 (myeloid leukaemia), H1975 (nonsmall lung cancer), SGC-7901 (gastric cancer), A549 (lung carcinoma), MOLT-4 (acute lymphoblastic leukemia) and HL-60, with IC_50_ values ranging from 0.19 to 35.4 μM. Moreover, **49** displayed a moderate antitubercular activity with an IC_50_ value of 17.5 μM [30].

**Figure 5:**
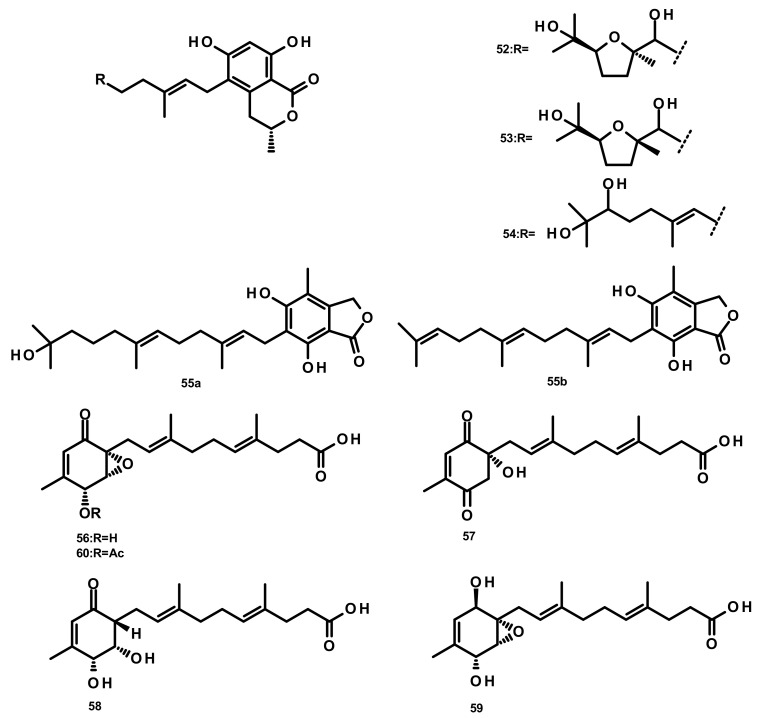
Chemical structures of acyclic sesquiterpenes **52**–**60**.

Verruculides B_2_ (**52**), B_3_ (**53**) and B (**54**) (Figure 5) were isolated from the fungus *Penicillium* sp. SCS-KFD09 which was obtained from a marine worm, *Sipunculus nudus*. Compound **52** showed weak antibacterial activity against *S. aureus*, with a MIC value of 32 μg/mL [31]. A farnesyl phthalide derivative **55a** and its previously reported analogue **55b** (Figure 5) were isolated from the marine sediment-derived fungus *Penicillium rudallense*. Compound **55a** did not significantly suppress receptor activator of nuclear factor κB ligand (RANKL)-induced osteoclast differentiation [32]. Five farnesylcyclohexenone derivatives, peniginsengins A–E (**56**–**60**) (Figure 5), were isolated from the culture extract of *Penicillium* sp. YPGA11 which was obtained from deep-sea water at a depth of 4500 m in the Yap Trench (West Pacific Ocean). The absolute configurations of their stereogenic carbons were established by comparison of the calculated and experimental electronic circular dichroism (ECD) spectra. Compounds **57**–**60** displayed weak to moderate antibacterial activity against *S. aureus* ATCC 25,913 and *S. aureus* ATCC 43,300 with MIC values of 8–32 μg/mL [33].

#### 2.3.2. Meroterpenoids Containing Monocyclic Sesquiterpenes (Figure 6)

**Figure 6:**
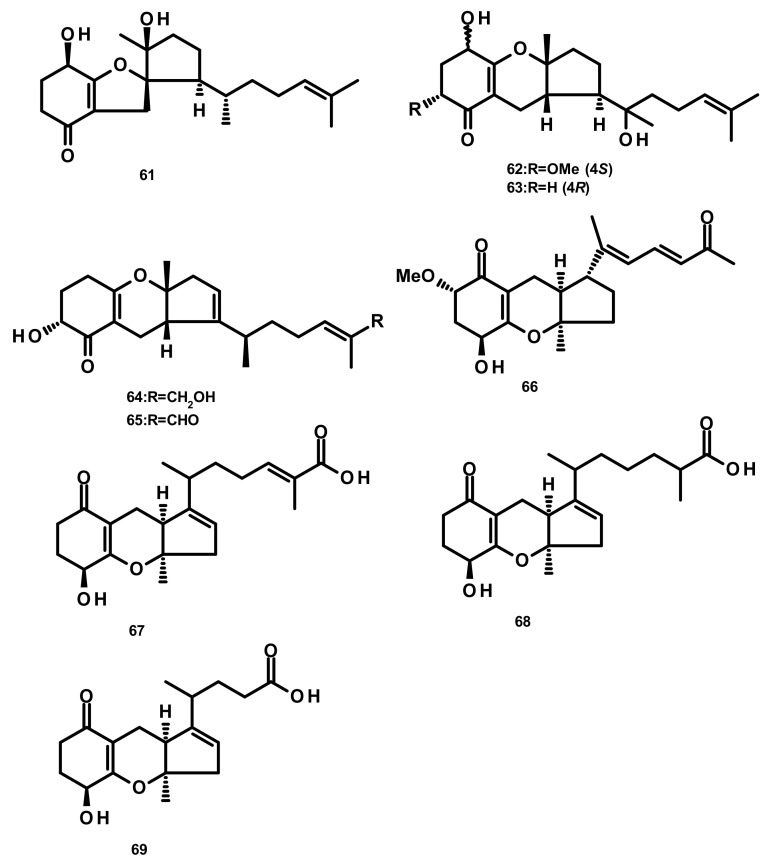
Chemical structures of monocyclic sesquiterpenes **61**–**69**.

Merosesquiterpenes containing a cyclopentane ring **61**–**65** (Figure 6) were obtained from *Alternaria* sp. JJY-32, which was isolated from the marine sponge *Callyspongia* sp. A shikimate-isoprenoid hybrid biosynthetic pathway for these compounds were proposed based on supplementation experiments with specific enzyme inhibitors and putative precursors. Compounds **61**–**65** displayed NF-κB inhibitory activities, in RAW264.7 cells, with IC_50_ values of 52, 76, 75, 50 and 39 μM, respectively [28]. Guignardone L (**66**) (Figure 6), also reported from the mangrove endophytic fungus *A. flavipes,* showed no significant antibacterial or cytotoxic activities [27]. Chemical examination of the culture extract of *Alternaria alternata* ICD5-11, which was obtained from a marine isopod *Ligia exotica*, led to the isolation of the previously described tricycloalternarene acid 11a (TCA 11a, **67**) and two previously unreported tricycloalternarenes K (**68**) and L (**69**) (Figure 6). Compounds **68** and **69** displayed no antibacterial activity against *Bacillus subtilis* and *S. aureus* by the disk diffusion method at a concentration of 20 μg/disk [34].

#### 2.3.3. Meroterpenoids Containing Bicyclic Sesquiterpenes

##### Drimane Sesquiterpenes Linked to a 2-pyrone (Figure 7)

**Figure 7:**
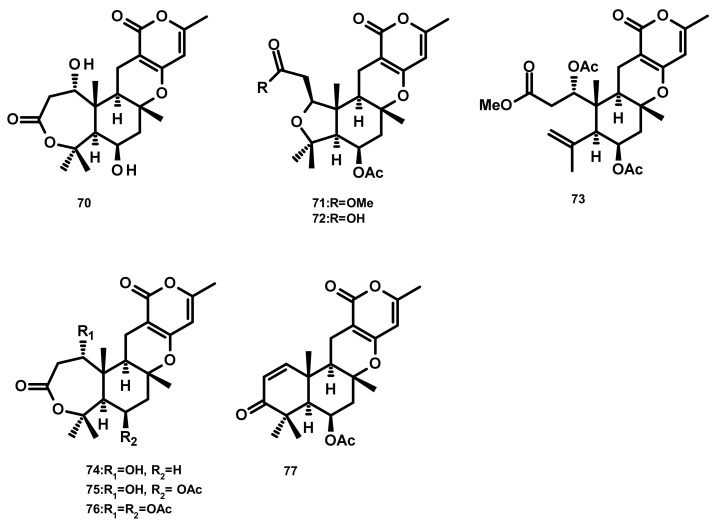
Chemical structures **70**–**77**.

Asperdemin (**70**) (Figure 7), a bicyclic merosesquiterpene, was isolated from the culture extract of the marine fungus *Aspergillus versicolor*, isolated from benthos of the Sakhalin Bay. Compound **70** displayed weak cytostatic and membranolytic activities in developing embryos of the sea urchin *Strongylocentrotus nudus* at a concentration of 6.38 mmol/L, and also induced haemolysis of human erythrocytes with an EC_50_ = 1.15 mmol/L [35]. Asperversins A (**71**) and B (**72**), merosesquiterpenes possessing a 5/6/6/6 ring system, and their analogues asperversins C–G (**73**–**77**) (Figure 7) featuring a 7/6/6/6 cyclic ring system, were isolated together with **70**, from the marine-derived fungus *Aspergillus versicolor*, isolated from the mud of the South China Sea. Compounds **70**–**77** exhibited neither cytotoxicity against A549, MCF-7, HepG2 (hepatocellular carcinoma) and HL-60 cell lines nor antibacterial activity against methicillin-resistant *S. aureus* (MRSA), methicillin-sensitive *S. aureus* (MSSA), *Escherichia coli* and *Pseudomonas aeruginosa.* Compounds **70**–**77** were also assayed for their AChE inhibitory activity; however only **77** showed a strong inhibitory activity with an IC_50_ value of 13.6 μM whereas the rest of the compounds was inactive at a concentration as high as 40 μM [36].

##### Drimane Sesquiterpenes Linked to a Phenyl 2-pyrone (Figure 8)

**Figure 8:**
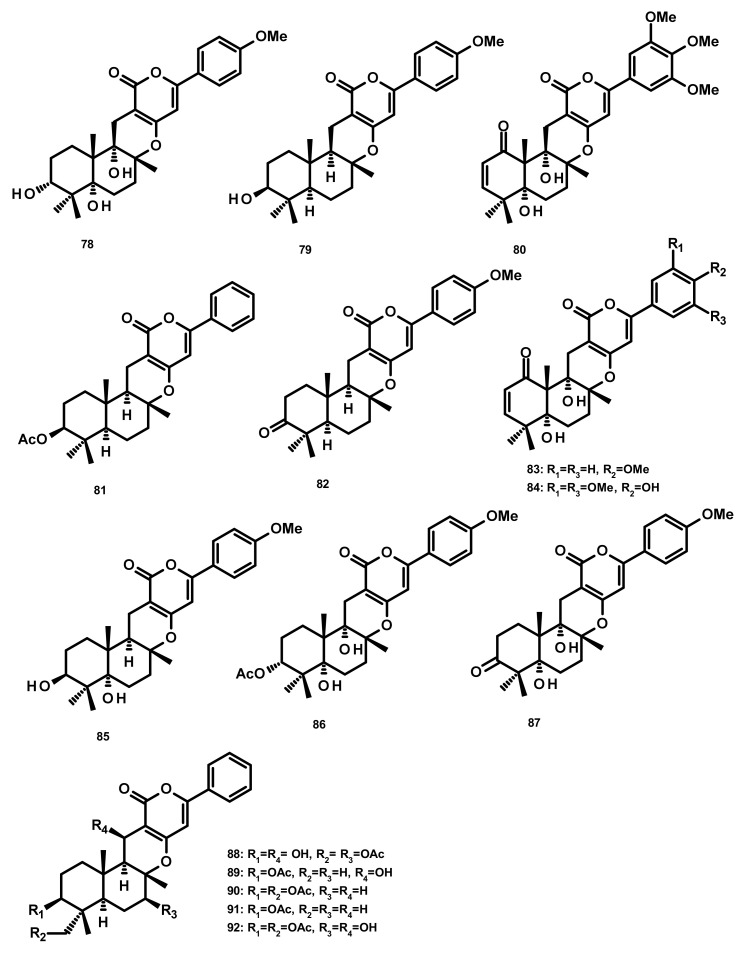
Chemical structures of **78**–**92**.

Three merosesquiterpenes containing a phenyl α-pyrone, arigsugacin I (**78**), arigsugacin F (**79**) and territrem B (**80**) (Figure 8), were isolated from the endophytic fungus *Penicillium* sp. sk5GW1L, which was obtained from the leaves of the mangrove plant *Kandelia candel*. Compounds **78**–**80** displayed a potent in vitro inhibitory activity against AChE, with IC_50_ values of 0.64 ± 0.08 µM, 0.37 ± 0.11 µM, and 7.03 ± 0.20 nM, respectively [37]. Another merosesquiterpene containing a phenyl α-pyrone, S14-95 (**81**) (Figure 8), was obtained from the marine sponge-associated fungus *Aspergillus similanensis* KUFA 0013, which was isolated from the marine sponge *Rhabdermia* sp. Compound **81** was evaluated for its antimicrobial activities against a panel of Gram-positive (*S. aureus* ATCC 25,923 and *B. subtilis* ATCC 6633) and Gram-negative bacteria (*E. coli* ATCC 25,922 and *P. aeruginosa* ATCC 27,853) and yeast (*Candida albicans* ATCC 10,231); however no activities were observed (MIC > 256 µg/mL) [38]. Arisugacin J (same as arigsugacin I, **78**), arisugacin F (same as arigsugacin F, **79**), arisugacin G (**82**), arisugacin B (**83**), territrem C (**84** (Figure 8)) and territrem B (**80**) were obtained from the endophytic fungus *Penicillium* sp. SXH-65, which was isolated from the leave of *Tamarix chinensis*. Compounds **82** and **83** exhibited weak cytotoxicity toward HL-60, K562 and Hela cell lines with IC_50_ values of 24.2, 36.2, 59.9 and 45.9, 46.6, 44.4 μM, respectively [29]. Chemical examination of the mangrove-derived fungus *Penicillium* sp., isolated from the leaves of the mangrove plant *Kandelia candel*, yielded 3-*epi*-arigsugacin E (**85**) arisugacin D (**86**), terreulactone C (**87**) (Figure 8) and the previously mentioned arisugacin B (**83**) and territrem C (**84**). Compounds **83**–**85** displayed potent AChE inhibitory activity with IC_50_ values of 3.03, 0.23 and 0.028 μM, respectively [39]. Ding et al. [40] reported the isolation of two previously unreported phenylpyropenes E (**88**) and F (**89**), together with the previously described phenylpyropenes B (**90**), C (**91**), and D (**92**) (Figure 8), from the culture of the marine-derived fungus *Penicillium concentricum* ZLQ-69 which was isolated from the water samples taken from the coast of the Bohai Sea in Binzhou, Shandong Province, China. Compounds **88**–**92** were assayed for their in vitro cytotoxicity against three human cancer cell lines, A549, MGC-803 (gastric cancer) and HL-60 by the MTT method. However, only **88** and **92** exhibited moderate cytotoxicity against the MGC-803 cell line with IC_50_ values of 19.1 and 13.6 μM, respectively.

##### Pyripyropenes (Figure 9)

**Figure 9:**
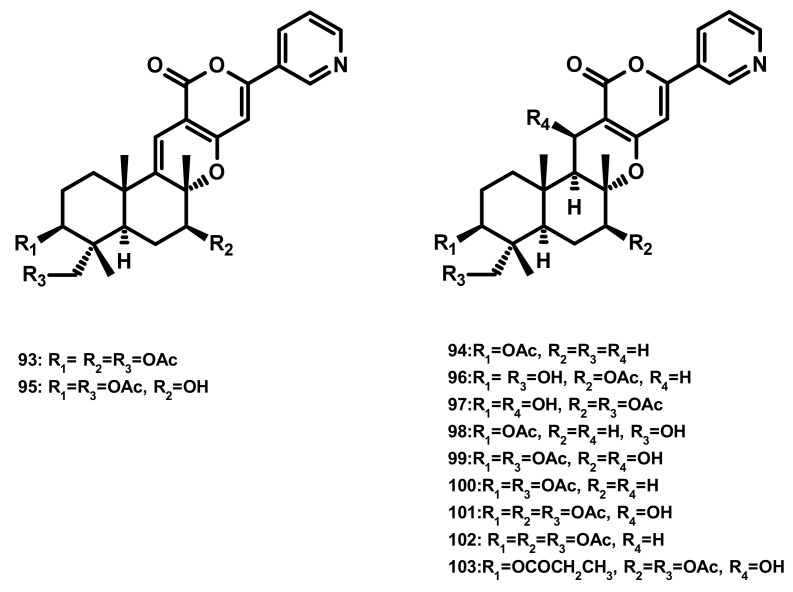
Chemical structures of pyripyropenes **93**–**103**.

Pyripyropenes S (**93**) and E (**94**), and the previously unreported pyripyropene T (**95**) (Figure 9) were isolated from the marine sponge-associated fungus *Aspergillus similanensis* KUFA 0013. Compounds **93**, **94** and **95** showed neither antibacterial nor antifungal activities against a panel of Gram-positive (*S. aureus* ATCC 25,923 and *B. subtilis* ATCC 6633) and Gram-negative bacteria (*E. coli* ATCC 25,922 and *P. aeruginosa* ATCC 27,853) and yeast (*C. albicans* ATCC 10,231) with MIC > 256 µg/mL [38,41]. Besides two previously unreported pyripyropene derivatives, 13-dehydroxy-1,11 deacetylpyripyropene A (**96**) and 1-deacetylpyripyropene A (**97**) (Figure 9), six previously described pyripyropenes, namely **94**, 11-deacetylpyripyropene O (**98**), 7-deacetylpyripyropene A (**99**), pyripyropenes O (**100**) and A (**101**) and 13-dehydroxypyripyropene A (**102**) (Figure 9) were isolated from the marine-derived fungus *Fusarium lateritium* 2016F18-1, which was isolated from the marine sponge *Phyllospongia foliascens*. Compounds **98**, **100** and **101** exhibited significant cytotoxicity against five human cancer cell lines, i.e., CNE1 (nasopharyngeal carcinoma), CNE2 (nasopharyngeal carcinoma), HONE1 (nasopharyngeal carcinoma), SUNE1 (spectrin repeat containing nuclear envelope protein 1), GLC82 (lung carcinoma) cancer cell lines as well as HL7702 (normal hepatic) cells [42]. Compounds **99**, **101** and **102** were also isolated from the marine-derived fungus *Neosartorya pseudofischeri* which was obtained from the inner tissue of the starfish *Acanthaster planci*. Compounds **99**, **101** and **102** exhibited significant cytotoxicity against Sf9 cells, highlighting them for a prospective platform for biorational pesticides development [43]. Compounds **94**, **100**, **101** and pyripyropene B (**103**) (Figure 9) were reported from the marine-derived fungus *Penicillium concentricum* ZLQ-69; however they were not assayed for any bioactivity [40].

##### Drimane Sesquiterpenes Linked to a 4-pyrone (Figure 10)

**Figure 10:**
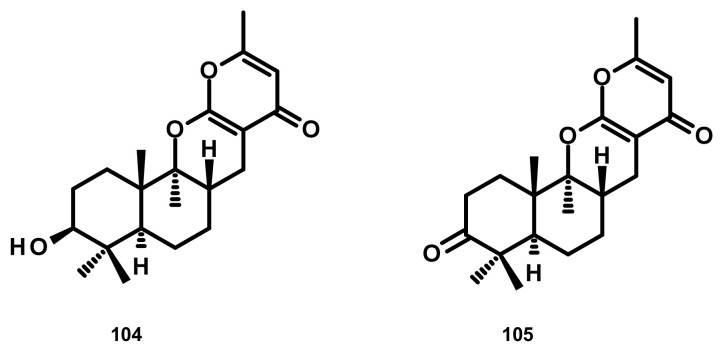
Chemical structures of **104** and **105**.

Penicillipyrones A (**104**) and B (**105**) (Figure 10), two merosesquiterpenes containing a 4-pyrone moiety, were isolated from the marine-derived fungus *Penicillium* sp. F446, which was obtained from marine sediments at the depth of 25 m collected from Geomun-do (Island), Korea. Compound **104** exhibited significant induction of quinone reductase in a dose-dependent manner in murine Hepa 1c1c7 (murine hepatoma) cells over the concentration range 5–40 μM [44].

##### Drimane Sesquiterpenes Linked to a Cyclohexanone Derivative by a Methylene Bridge (Microphorin-Related Compounds) (Figure 11)

**Figure 11:**
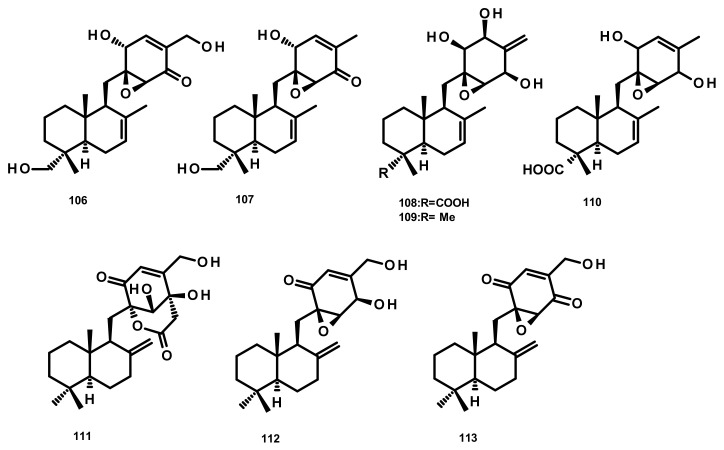
Chemical structures of **106**–**113**.

Epoxyphomalins A (**106**) and B (**107**) (Figure 11) were isolated from the culture extract of the marine-derived fungus *Paraconiothyrium* cf. *sporulosum* (initially identified as *Phoma* sp. 193H12), obtained from the marine sponge *Ectyplasia perox,* which was collected from the Caribbean Sea. Compounds **106** and **107** displayed significant antiproliferative activity against a panel of 36 human tumour cell lines with IC_50_ values of 0.11 and 1.25 µg/mL, respectively [45]. Further investigation of the same fungus by the same research group has resulted in the isolation of three new analogues which were named epoxyphomalins C (**108**), D (**109**) and E (**110**) (Figure 11). Compounds **108**–**110** were evaluated for their cytotoxicity against the same panel of 36 tumour cell lines. Although **108** and **110** were inactive at a concentration of 27.6 μM, **109** displayed a selective cytotoxicity, particularly against the prostate PC3M and bladder BXF 1218 L cancer cell lines with IC_50_ values of 0.72 and 1.43 μM, respectively [46]. Purpurogemutantin (**111**), macrophorin A (**112**) and 4′-oxomacrophorin (**113**) (Figure 11), three merosesquiterpenes featuring a quinone/hydroquinone scaffold, were isolated from the marine sponge-associated fungus *Gliomastix* sp. ZSDS1-F7, which was obtained from the marine sponge *Phakellia fusca* collected on the Yongxing island of Xisha, China. Compounds **111**–**113** showed significant in vitro cytotoxicity against various cancer cell lines including K562, MCF-7, Hela, DU145, U937, H1975, SGC-7901, A549, MOLT-4 and HL-60, with IC_50_ values ranging from 0.19 to 35.4 μM. Moreover, **112** and **113** displayed a moderate antitubercular activity with IC_50_ values of 22.1 and 2.44 μM, respectively [30].

##### Drimane Sesquiterpenes Linked to Spirobezopyran Derivatives (Figure 12)

**Figure 12:**
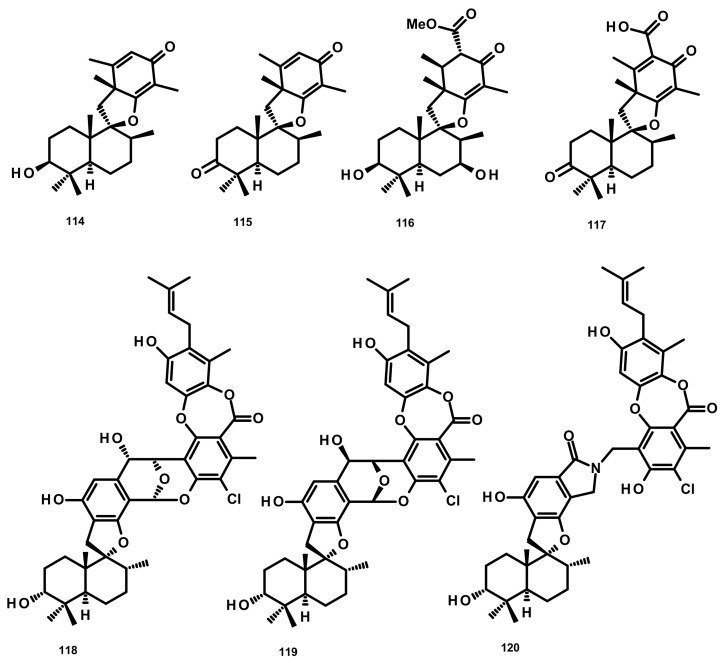
Chemical structures of **114**–**120**.

Chermesins A-D (**114**–**117**) (Figure 12), three spiropyranoquinone drimanes, were isolated from the culture extract of the algicolous fungus *Penicillium chermesinum* EN-480 which was obtained from the marine red alga *Pterocladiella tenuis*. Compounds **114**–**117** were assayed for their antimicrobial activity against four human pathogenic bacteria (*E. coli, Micrococcus luteus* and *P. aeruginosa*) and yeast (*C. albicans*), five plant pathogenic fungi (*Alternaria brassicae, Colletotrichum gloeosporioides, Fusarium oxysporum, Gaeumannomyces graminis,* and *Physalospora piricola*) and five aquatic bacteria (*Aeromonas hydrophila*, *Edwardsiella tarda*, *Vibrio alginolyticus, V. harveyi, and V. parahemolyticus*). Compounds **114** and **115** were active against *C. albicans, E. coli, M. luteus*, and *V. alginolyticus*, with MIC values ranging from 8 to 64 μg/mL, whereas **117** only showed weak activity against *E. coli* (MIC = 64 μg/mL). Compound **116** exhibited no activity against all of the tested strains [47]. Chartarolides A–C (**118**–**120**) (Figure 12), another spirobenzopyran drimanes isolated from the fermentation broth of the marine-derived fungus *Stachybotrys chartarum* WGC-25C-6, which was obtained from the marine sponge *Niphates recondite*, exhibited significant cytotoxicity against a panel of human tumour cell lines including HCT-116 (colon carcinoma), HepG2, BGC-823 (gastric carcinoma), NCI-H1650 (nonsmall cell lung carcinoma), A2780 (ovarian carcinoma) and MCF-7, with IC_50_ values ranging from 1.4 to 12.5 μM. These compounds also exhibited durable inhibitory activities against four tumour-associated protein kinases, including FGFR3, IGF1R, PDGFRb and TrKB, with IC_50_ values ranging from 2.6 to 25 μM [48].

##### Drimane Sesquiterpenes Linked to Isochromone Derivatives (Figures 13 and 14)

**Figure 13:**
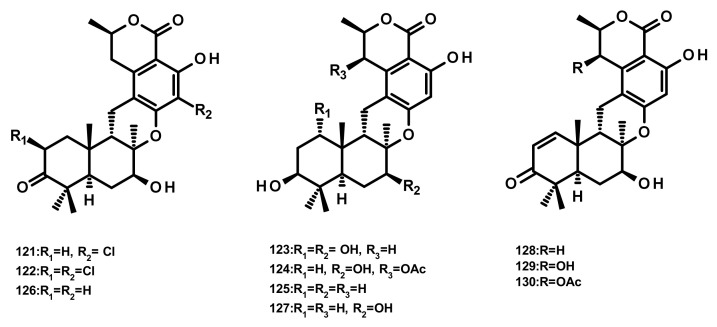
Chemical structures of **121**–**130**.

Ten isochromone-based drimanes, including chrodrimanins K (**121**), L (**122**), M (**123**), N (**124**), H (**126**), F (**127**), E (**128**), A (**129**) and B (**130**) and hydroxypentacecilide A (**125**) (Figure 13), were isolated from the fermentation broth of *Penicillium* sp. SCS-KFD09 which was obtained from a marine worm *Sipunculus nudus*. Compounds **121**, **124** and **125** (Figure 13) displayed weak antiviral activity against the influenza A virus (H1N1) with IC_50_ values of 74, 58, and 34 μM, respectively [31]. Compounds **129** and **130** were also isolated from the culture extract of the endophytic fungus *Talaromyces amestolkiae* YX1, obtained from healthy leaves of the mangrove tree *Kandelia obovata*, collected from Zhanjiang Mangrove Nature Reserve in Guangdong, China [20].

**Figure 14:**
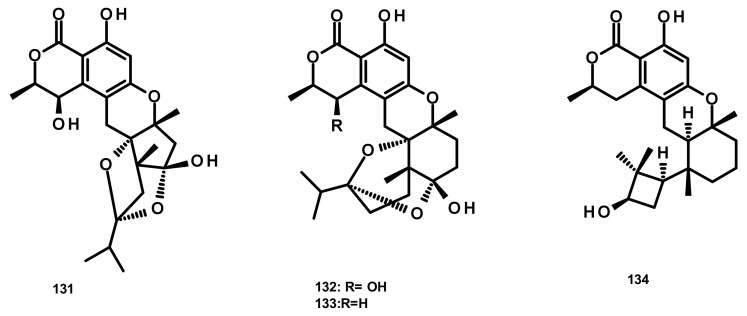
Chemical structures of **131**–**134**.

Talaromyolides A-D (**131**–**134**) (Figure 14), analogs of seco-drimane linked to isochromone, were isolated from the marine-derived fungus *Talaromyces* sp. CX11, obtained from the red seaweed *Grateloupia filicina*. Compounds **131**–**134** displayed no cytotoxicity against a panel of human tumour cell lines including HL-60, K562, MGC-803, BEL-7402 (hepatocellular carcinoma), SH-SY5Y (neuroblastoma), HCT-116, MDA-MB-231 (triple negative breast cancer), A549, MCF-7/ADM (Adriamycin-resistant breast cancer), HO8910 (ovarian cancer), U87 (glioblastoma) and NCI-H1975 (nonsmall cell lung cancer). Interestingly, **134** exhibited potent antiviral activity against pseudorabies virus (PRV) with a CC_50_ value of 3.35 μM [19].

##### Drimane Sesquiterpenes Linked to 5-Methylorsellinic Acid

###### Austalides (Figure 15)

**Figure 15:**
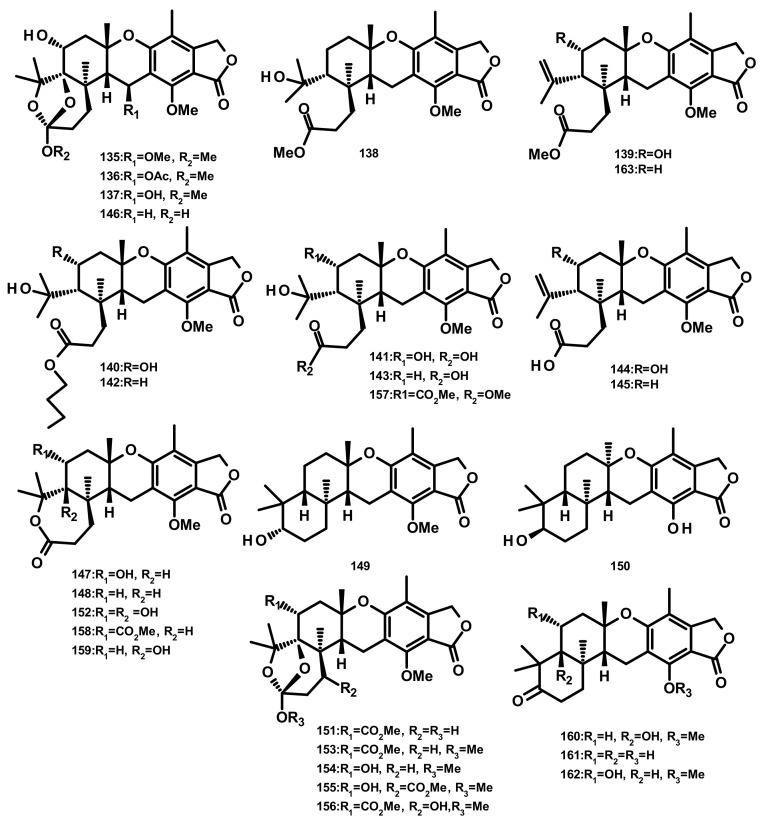
Chemical structures of australides **135**–**163**.

Five austalides M-Q (**135**–**139**) (Figure 15) were isolated from the marine-derived fungus *Aspergillus* sp. which was obtained from the Mediterranean sponge *Tethya aurantium*. Compounds **135**–**139** were evaluated for their cytotoxic activity against the murine cancer cell line L5178Y by the MTT assay; however, none of them were active [49]. Australide H acid butyl ester (**140**), australide H acid (**141**), australide P acid butyl ester (**142**), australide P acid (**143**), australide Q acid (**144**), 13-*O*-deacetylaustalide I (**147**) and 13-deacetoxyaustralide I (**148**) (Figure 15) were isolated from the culture extract of *Penicillium thomii* KMM 4645 whereas **141–144**, **147, 148**, 13-deoxyaustralide Q acid (**145**), 17-*O*-demethylaustalide B (**146**) and 17*S*-dihydroaustalide K (**149**) (Figure 15) were isolated from the culture of *P. lividum* KMM 4645. Both of the fungal strains were isolated from the superficial mycobiota of the brown alga *Sargassum miyabei*, collected from the Sea of Japan [50]. Compounds **140**, **141**, **146,** and **148** did not exhibit cytotoxicity against MDA-MB-231 and JB6 Cl41 (mouse epidermal) cell lines; however, these compounds inhibited AP-1-dependent transcriptional activity in JB6 Cl41 cell line at noncytotoxic concentrations after 12 h of treatment. Interestingly, these compounds showed significant inhibitory activity at a concentration of 6.25 μM but did not reduce cell viability at 100 μM [50]. Moreover, **140**–**144**, **147**, and **148** exhibited strong inhibitory activity against *endo*-1,3-β-d-glucanase, obtained from a crystalline stalk of the marine mollusk *Pseudocardium sachalinensis* [50]. The culture extract of the marine sponge-associated fungus *Aspergillus aureolatus* HDN14-107, isolated from an unidentified sponge, furnished **143**, **147**, austalides S-U (**150**–**152**), A (**153**), B (**154**), D (**155**), E (**156**), G (**157**), I (**158**), J (**159**), L (**160**) (Figure 15). Compounds **152** and **154** displayed an antiviral activity against the influenza A virus (H1N1), with IC_50_ values of 90 and 99 µM, respectively [18]. Austalides V-X (**161**–**163**) (Figure 15) were isolated, together with **138**, **139**, **143**, **148**, **149** and **160**, from the marine sediment-derived fungus *Penicillium rudallense*. Compounds **161** and **162** displayed significant inhibitory activity on RANKL-induced osteoclast differentiation with ED_50_ values of 1.9–2.8 μM [32].

##### Drimane Sesquiterpenes Linked to 3,5-Methylorsellinic Acid

###### Austinoids and Related Compounds (Figures 16–18)

**Figure 16:**
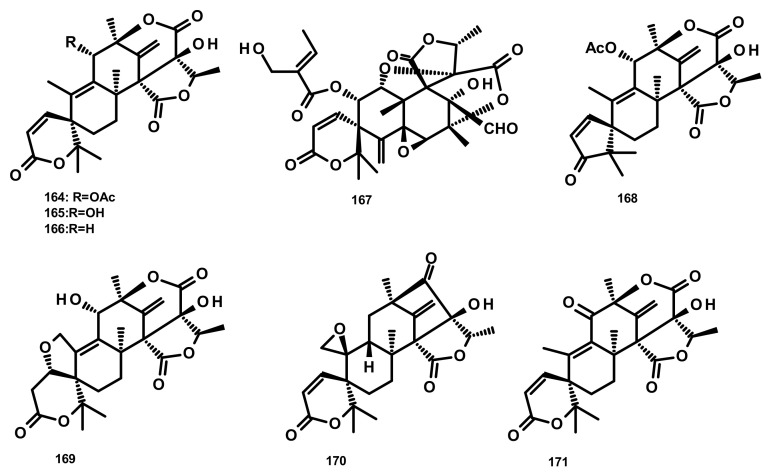
Chemical structures of austinoids **164**–**171**.

**Figure 17:**
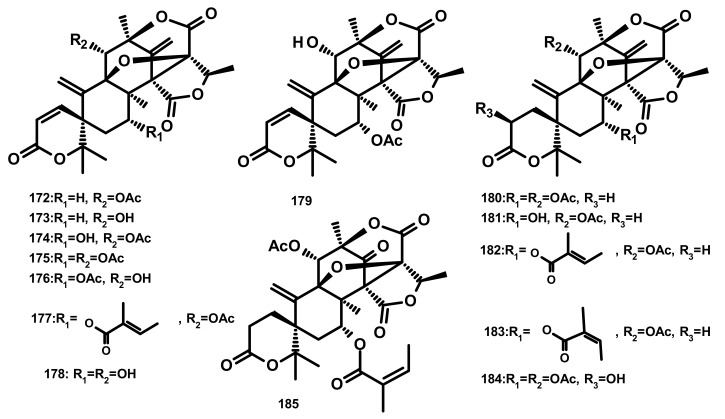
Chemical structures dehydroaustinoid derivatives **172**–**185**.

**Figure 18:**
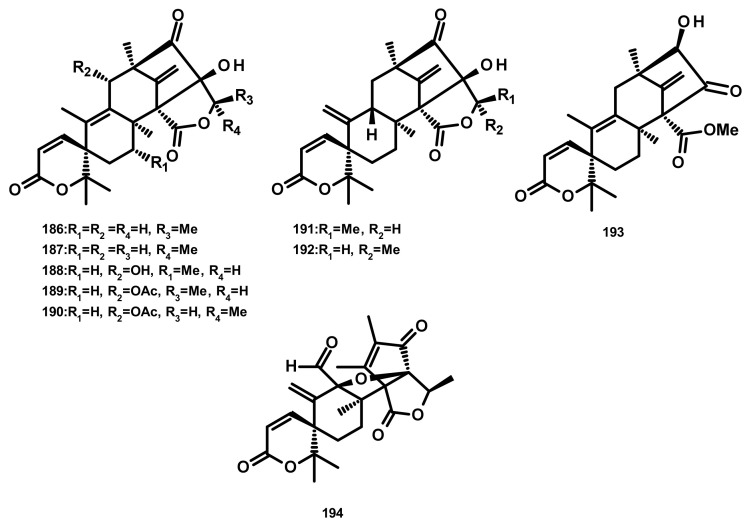
Chemical structures isoaustinoids **186**–**194**.

Merosesquiterpenes of the austinoid group can be arbitrarily divided, according to their structural variances, into austinoids (Figure 16), dehydroaustinoids (Figure 17) and isoaustinoids (Figure 18). For practical aspects of the discussion of these compounds, they will not be categorized into any particular group.

Austinol (**165**) (Figure 16), dehydroaustin (**172**) and 11α-acetoxyisoaustinone (**189**) (Figure 18) were isolated from the culture extract of *Penicillium citrinum*, which was obtained from the mangrove *Bruguiera sexangula* var. *rhynchopetala*, collected in the South China Sea. Compounds **165**, **172** and **189** showed selective antibacterial activity against five terrestrial and two marine pathogenic bacteria, particularly **165** displayed moderate activity against *Staphylococcus epidermidis* and *S. aureus* with MIC values of 10 µM. However, these compounds showed no cytotoxicity (IC_50_ > 50 µM) against HeLa, MCF-7 and A549 cell lines [51]. Talaromytin (**167**) (Figure 16), isolated from the marine seaweed-derived fungus *Talaromyces* sp. CX11, exhibited no cytotoxicity against a panel of human tumour cell lines, including HL-60, K562, MGC-803, BEL-7402, SH-SY5Y, HCT-116, MDA-MB-231, A549, MCF-7/ADM, HO8910, U87 and NCI-H1975 [19]. Further structurally related austinoids, namely austin (**164**), **172**, dehydroaustinol (**173**), 7-hydroxydehydroaustin (**174**), acetoxydehydroaustin (**175**) (Figure 17) and **189**, were isolated from the marine-derived fungus *Pestalotiopsis* sp. PSU-ES194, which was obtained from leaves of the seagrass *Enhalus acoroides.* Compound **175** displayed weak cytotoxicity against Vero cells with an IC_50_ value of 48 μM [52]. Long et al. [21] described the isolation of **164**, **165**, **172**, **173**, **175**, 1, 2-dihydroacetoxydehydroaustin (**180**), 2-hydroacetoxydehydroaustin (**184**) (Figure 17), isoaustinone (**186**) (Figure 18), and **189**, from the culture of the fungus *Aspergillus* sp. 16–5c, which was obtained from leaves of the mangrove plant *Sonnera tiaapetala*, collected on the coastal salt marsh of the South China Sea. Compounds **172**, **173** and **186** exhibited inhibitory activity against AChE with IC_50_ values of 0.40, 3.00 and 2.50 µM, respectively. Later on, Liu et al. described the isolation of a new austinoid derivative, 1,2-dehydroterredehydroaustin (**183**) (Figure 17), together with the previously reported **175** and **180**, from *Aspergillus terreus* H010, which was isolated from the mangrove tree *Kandelia obovata*. The absolute configurations of the stereogenic carbons of **180** were determined by comparison of the calculated and experimental ECD spectra. Compound **180** exhibited weak anti-inflammatory activity with an IC_50_ value of 42.3 μM [53] (Figure 16 and Figure 17).

Two previously unreported austinoid derivatives, furanoaustinol (**169**) (Figure 16) and 7-acetoxydehydroaustinol (**176**) (Figure 17) were isolated, along with **164**, **165**, austinolide (**166**) (Figure 16), (**172**), 7-hydroxydehydroaustin (**174**) (Figure 17), **175**, 11α-hydroxyisoaustinone (**188**) (Figure 18) and **189**, from the culture of the marine-derived fungal strain *Penicillium* sp. SF-549, collected from a sample of sea sand. Compound **169** showed weak inhibitory activity against protein tyrosine phosphatase 1B with an IC_50_ value of 77.2 μM, whereas **166, 175**, **176**, **188**, and **189** showed weak inhibition of NO production with IC_50_ values of 30.1, 58.3, 61.0, 37.6, and 40.2 μM, respectively [54]. The culture extract of *Penicillium* sp. TGM112, isolated from the medicinal mangrove *Bruguiera sexangula* var. *rhynchopetala*, collected in the South China Sea, afforded two previously unreported austin analogs, penicianstinoids A (**185**) and B (**178**), in addition to the previously described **164**, **165**, **169**, **173**, **174**, 1, 2-dihydro-7-hydroxydehydroaustin (**181**) and **189**. The absolute configurations of the stereogenic carbons of **178** and **185** were determined by comparison of the experimental and calculated ECD spectra using Time-Dependent Density-Functional Theory (TDDFT), while those of **169** and **181** were confirmed by X-ray analysis. Compounds **164**, **165**, **178** and **185** displayed growth inhibitory activity of newly hatched larvae of cotton bollworm (*Helicoverpa armigera* Hubner) with IC_50_ values of 200 µg/mL (the positive control azadirachtin; IC_50_ = 25 μg/mL). Compounds **165**, **169**, **173**, **174**, **178**, **181** and **185** exhibited insecticidal activity against a nematode *Caenorhabditis elegans* with EC_50_ values ranging from 9.4 (± 1.0) to 38.2 (± 0.6) μg/mL [55]. Hwang et al. [56] reported the isolation of the previously reported austinoids, including **164**, **172**, **173**, **175**, **186**, 5′*S*-isoaustinone (**187**), **189**, neoaustin (**191**) and austinoneol A (**193**) (Figure 18), from the culture extract of the marine-derived fungus *Penicillium* sp. FCH061, isolated from the underwater sediment collected off the coast of Chuja-do in Korea. It is worth mentioning that the stereostructures of these compounds in the reference are opposite to those described in this review. Asperaustins A (**168**) (Figure 16) and B (**190**) (Figure 18) were obtained, together with the previously described austinoids, i.e., **164**, **172**, precalidodehydroaustin (**177**), **186**, **187**, **193**, from the culture extract of *Aspergillus* sp. ZYH026, isolated from superficial mycobiota of the brown alga *Saccharina cichorioides* f. *sachalinensis*, which was collected from the South China Sea [57] (Figure 17). The absolute structures of **168**, **177**, **190** and **193** were established unambiguously by single-crystal X-ray analysis using CuKa radiation. All the isolated compounds, except **168**, were assayed for AChE inhibitory activity but none exhibited significant activity [57].

The previously unreported austinoid derivatives, brasilianoids G (**194**) (Figure 18), H (**170**), I (**171**) J (**179**) and L (**192**) were reported, together with the previously described austinoids including **165**, **166**, **169**, **172**, **173**, **174**, **188** and **191**, from the marine-derived fungus *Penicillium brasilianum* WZXY-M122-9, isolated from a marine sponges collected from the South China Sea. None of the isolated compounds exhibited either cytotoxicity against A549, RAW264.7 (mouse monocyte/macrophage) and IEC-6 (rat small intestine epithelial) cell lines or antibacterial activity against Gram-positive bacteria *S. aureus* ATCC 29,213 and a clinically isolate Gram-negative bacteria *Klebsiella pneumoniae* 58AP) [58] (Figure 18).

##### Preaustinoids and Related Compounds (Figures 19 and 20)

**Figure 19:**
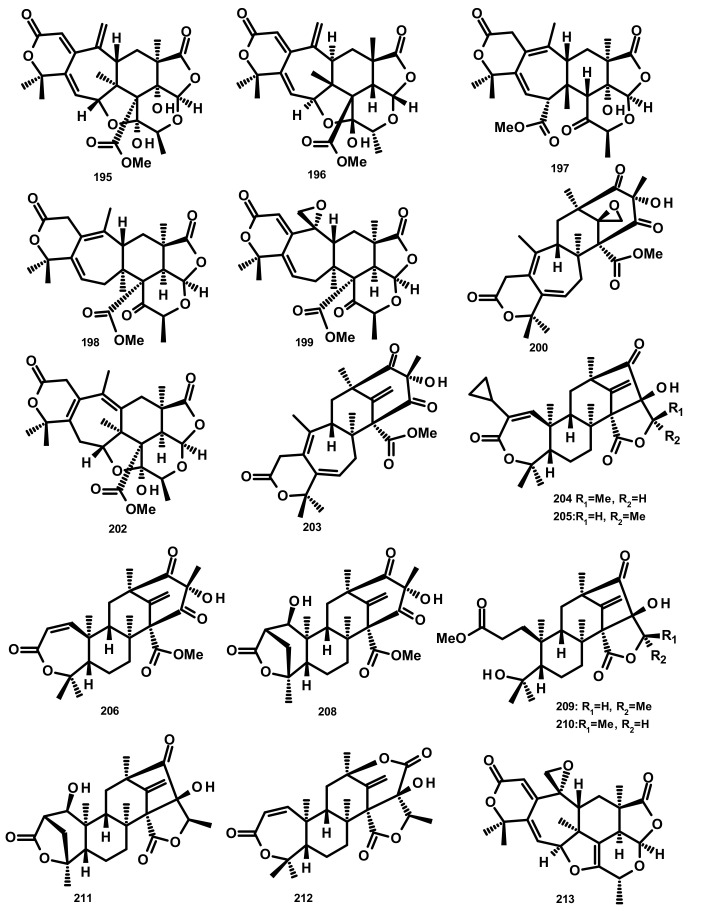
Chemical structures of preaustinoids **195**–**200**, **202**–**206** and **208**–**213**.

**Figure 20:**
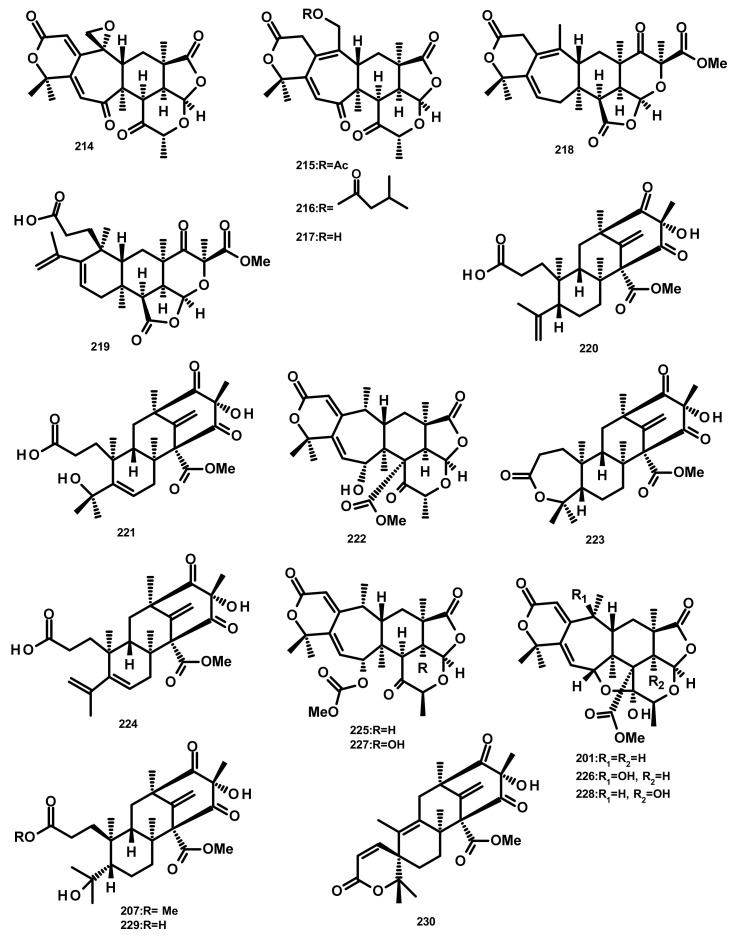
Chemical structures of preaustinoid derivatives **201**, **207** and **214**–**230**.

Zhang et al. [59] reported the isolation of three new preaustinoids named 4,25-dehydrominiolutelide B (**195**), 4,25-dehydro-22-deoxyminiolutelide B (**196**) and isominiolutelide A (**197**), together with the previously reported berkeleyacetal A (**198**), berkeleyacetal B (**199**) and 22-epoxyberkeleydione (**200**) (Figure 19) from a static culture of the fungus *Penicillium* sp. MA-37, isolated from the rhizospheric soil of the mangrove plant *Bruguiera gymnorrhiza*, collected from the Hainan island. The absolute configurations of the stereogenic carbons of **195** and **197** were established by comparison of the experimental and calculated ECD spectra using TDDFT, whereas those of **196** were determined by single-crystal X-ray analysis using CuKa radiation. Li et al. [60] described the isolation of two new analogues of berkeleyacetal (which were later reisolated by Hoang et al. [61] and named 22-deoxyminiolutelide B (**201)** (Figure 20) and miniolutelide C (**202**) (Figure 19)), along with the previously reported **198**, **200** and berkeleydione (**203**) (Figure 19), from *Penicillium* strain 303#, obtained from sea water from Zhanjiang Mangrove National Reserve in Guangdong Province, China. Compounds **201** and **202** displayed moderate cytotoxicity against MDA-MB-435, HepG2, HCT-116, and A549 cell lines [60]. Two previously unreported preaustinoid analogs, preaustinoids E (**204)** and F (**205**), were isolated together with the previously reported preaustinoid A2 (**206**) (Figure 19) and preaustinoid D (**207**) (Figure 20) from the underwater sediment-derived fungus *Penicillium* sp. FCH061. The relative stereochemistry of **204** and **205** was determined by nuclear overhauser effect spectroscopy (NOESY) correlations and compared with that of the previously reported compounds [56]. Zhang et al. [62] described the isolation of six new preaustinoid derivatives namely brasilianoids A (**208**), B (**204**), C (**205**), D (**209**), E (**210**) and F (**211**) (Figure 19), together with the previously reported **206** and **207**, from a marine-derived fungus *P. brasilianum* WZXY-m122-9, isolated from an unidentified sponge. Surprisingly, the structures of brasilianoids B (**204**) and C (**205**) are found to be the same as those of preaustinoids E (**204**) and F (**205**), previously isolated by Wang et al. [56], although the stereostructures of **204** and **205** in the original article [56] are opposite to those of brasilianoid B (**204**) and C (**205**). Compound **208** significantly stimulated filaggrin and caspase-14 expressions in HaCaT (human keratinocyte) cells in a dose-dependent manner. Since filaggrin is a key natural moisturizing factor that maintains the ability to regulate the skin moisture barrier, **208** could be a potential cosmeceutical for skin moisturizer in the cosmetic industry. Moreover, **204** and **205** exhibited an inhibition of NO production in lipopolysaccharide (LPS)-induced RAW 264.7 macrophages whereas **204**–**206** (10 µM) inhibited the expression of the hepatitis B virus (HBV) DNA in HepG2.2.15 cells with the inhibitory rates of 25, 15, and 10%, respectively. Later on, the same group has reported a new preaustinoid, named brasilianoid K (**212**) (Figure 19) from the same fungus [58]. Chen et al. [20] reported the isolation of four new analogues of berkeleyacetals, amestolkolides A–D (**213**–**216**) (Figure 19 and Figure 20), along with the known analogue and purpurogenolide E (**217**) (Figure 20), from the culture extract of the endophytic fungus *Talaromyces amestolkiae* YX1, isolated from healthy leaves of the mangrove tree *Kandelia obovata*, collected from Zhanjiang Mangrove Nature Reserve in Guangdong, China. The absolute configurations of the stereogenic carbons of **213** and **216** were established by comparison of their calculated and experimental ECD spectra, whereas the stereostructures of **214** and **215** were established by a single-crystal X-ray diffraction analysis using CuKα radiation. Compounds **213** and **214** were tested for their anti-inflammatory activity by inhibition of the LPS-activated NO production in RAW264.7 cells for which **213** showed strong inhibitory activity with an IC_50_ value of (1.6 ± 0.1 μM) whereas **214** exhibited only weak activity, with an IC_50_ value of 30 ± 1.2 μM [20]. The organic extract of the culture extract of the marine sponge-associated fungus *Eupenicillium* sp. 6A-9, isolated from the inner tissue of the marine sponge *Plakortis simplex*, which was collected from Yongxing Island, China, furnished five new preaustinoids, namely eupeniacetals A (**218**) and B (**219**), preaustinoid A3 (**230**) (Figure 20), 1-methoxyhydropreaustinoid A1 (same as preaustinoid D) (**207**), hydroberkeleyone B (**221**) and 22-deoxy-10-oxominiolutelide B (**222**) (Figure 20), together with five previously reported preaustinoid derivatives including **198**, **201**, preaustinoid A1 (**223**) and berkeleyone C (**224**). All the isolated compounds, except **224** (Figure 20), exhibited inhibitory effects on tumour necrosis factor-α (TNF-α) secretion in LPS-induced THP-1 (leukemic monocyte) cells, with IC_50_ values ranging from 22.6 to 72.2 µM (pomalidomide, IC_50_ = 0.23 µM) [63]. Using the OSMAC (One Strain Many Compounds) approach and a metabolomic-oriented strategy, Hoang et al. [61] were able to identify and isolate two previously undescribed preaustinoids 22-deoxyminiolutelide A (**225**) and 4*S*-hydroxy-22-deoxyminiolutelide B (**226**), together with other previously reported preaustinoids including **198**, **201**, **202**, **218**, **222**, miniolutelide A (**227**) and miniolutelide B (**228**) (Figure 20), from the culture extract of the marine-derived fungus *Penicillium ubiquetum* MMS330, isolated from a sample of the blue mussel *Mytilus edulis*, collected at Port Giraud on the Loire estuary in France. All the isolated compounds, except **222**, were evaluated for their cytotoxicity against KB (keratin-forming tumour) and MCF-7 cell lines, however, neither of them exhibited significant activity. The previously undescribed preaustinoid derivatives, preaustinoids A6 (**229**) and A7 (**220**) (Figure 20) were reported, together with the previously described **206**, **224**, and preaustinoid A3 (**230**) (Figure 20), from the marine-derived fungus *Penicillium* sp. SF-5497. Compound **224** and **229** inhibited PTP1B (a member of the protein tyrosine phosphatase (superfamily) activity in a dose-dependent manner with IC_50_ values of 58.4 and 17.6 µM, respectively. Mechanistic study revealed that **201** inhibited PTP1B in a noncompetitive manner and preferentially bound to the free enzyme rather than to the enzyme-substrate complex [64]. Wen et al. [57] reported the isolation of a preaustinoid, named asperaustin C, from the algicolous fungus *Aspergillus* sp. ZYH026, which was claimed to be a new compound. The structure of asperaustin C, whose structure and absolute configurations of its stereogenic carbons were confirmed by X-ray analysis using Cu Kα radiation, was found to be the same as that of the previously described brasilianoid B (**204**). As the absolute configurations of the stereogenic carbons of preaustinoids E (**204)** and F (**205**), preaustnoid A2 (**206**) and preaustinoid D (**207**), reported by Hwang et al. [56], were determined by comparison with those described before the revision of the absolute configurations by Zhang et al. [62] and Wen et al. [57], the stereostructures of these compounds are opposite to those presented in this review.

##### Terretonins and Related Compounds (Figure 21)

**Figure 21:**
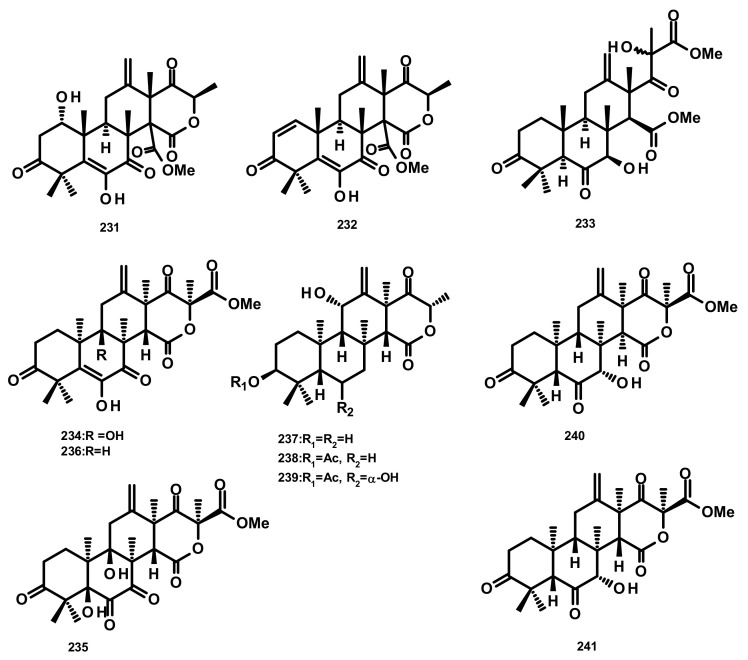
Chemical structures of terretonin derivatives **231**–**241**.

Terretonins E (**231**) and F (**232**) (Figure 21) were isolated from the culture extract of the marine derived-fungus *Aspergillus insuetus*, isolated from the marine sponge *Petrosia ficiformis* which was collected in the Mediterranean Sea. Compounds **231** and **232** inhibited NADH oxidase activity (in beef heart submitochondrial particles) with IC_50_ values of 3.90 ± 0.4 and 2.97 ± 1.2 µM, respectively [65]. The culture extract of the marine sponge-associated fungus *Aspergillus* sp. OPMF00272 furnished terretonin G (**233**) and terretonin (**234**) (Figure 21). Compound **233** (20 mg per 6 mm disk) exhibited antibacterial activity against Gram-positive bacteria (*S. aureus* FDA209P, *B. subtillis* PCI219 and *M. luteus* ATCC9341), but not against Gram-negative bacteria (*P. aeruginosa* IFO12689 and *E. coli* JM109) and yeast (*C. albicans* ATCC64548 and *S. cerevisiae* S288c) [66]. Chemical examination of the endophytic fungus *Aspergillus terreus* EN-539, obtained from the fresh tissue of the marine red alga *Laurencia okamurai* which was collected from the coast of Qingdao, China, led to the isolation of the previously unreported terretonin analogue, aperterpene O (**235**), together with the previously described terretonins A (**236**) (Figure 21) and G (**233**) [67]. Compound **233** exhibited antimicrobial activity against *M. luteus* and *S. aureus* with MIC values of 32 and 8 μg/mL, respectively [67]. A new terretonin analog terretonin O (**237**) was isolated, together with the previously reported terretonins M (**238**) and N (**239**) (Figure 21) from the culture extract of *Aspergillus terreus* LGO13, obtained from a sediment sample collected from sewage water containing heavy metals. Compound **237** displayed weak antimicrobial activity against *P. aeruginosa and S. aureus* [68]. The previously unreported terretonin D1 (**240**) (Figure 21) was isolated, together with the previously described **234**, **236** and terretonin D (**241**) (Figure 21), from the marine-derived fungus *Aspergillus terreus* ML-44, obtained from the fresh gut of pacific oyster. All the isolated compounds displayed a weak inhibition of NO production in the LPS-stimulated RAW264.7 macrophages [69]. It is interesting to note that only the relative configurations of the structures of **231**–**233** were determined. On the contrary, the absolute configurations of the stereogenic carbons of **237**–**241** were established, with absolute confidence, by X-ray analysis using CuKα radiation with good Flack parameter. Therefore, it is possible that the structures of **231**–**233** are the enantiomeric form of their correct structures.

##### Andrastins and Related Compounds (Figure 22)

**Figure 22:**
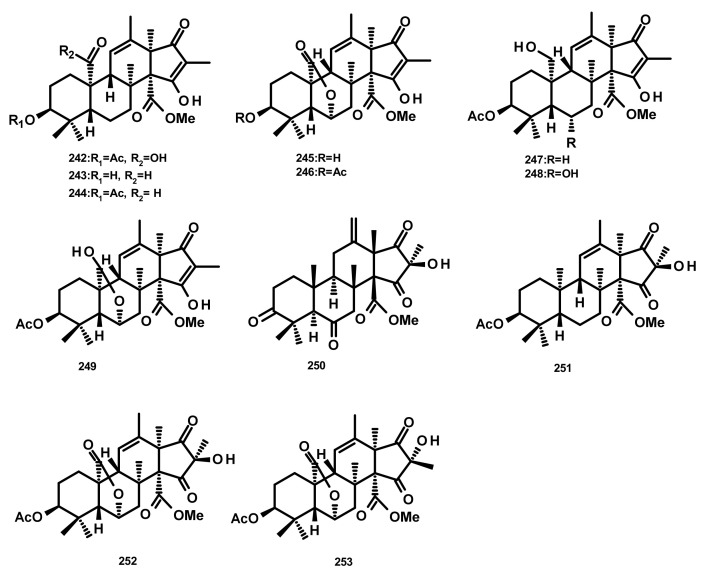
Chemical structures of andrastin derivatives **242**–**253**.

The andrastin derivatives, 15-deacetylated citreohybridone E (**242**), 3-deacetylated andrastin A (**243**), andrastin A (**244**), 3-deacetylcitreohybridonol (**245**), citreohybridonol (**246**), andrastin B (**247**), 6-*α*-hydroxyandrastin B (**248**) and dihydrocitreohybridonol (**249**) (Figure 22), were isolated from the marine-derived fungus *Penicillium* sp. YPGA11, obtained from the deep-sea water at a depth of 4500 m in the Yap Trench, West Pacific Ocean. Compounds **242**–**249** exhibited inhibitory activity against NO production in LPS-activated RAW 264.7 macrophages with inhibition rates ranging from 60% to 90% at 50 μM, but decreased sharply at 25 μM. Since these compounds were also cytotoxic to the RAW 264.7 cells (45–65% inhibition at 50 μM), it is believed that their inhibition of NO production was attributed to cell death [70]. Chemical examination of the algicolous fungus *Aspergillus terreus* EN-539 led to the isolation of another andrastin derivative named aperterpene N (**250**) [67]. Compound **250** (Figure 22) displayed the in vitro inhibitory activity against the influenza neuraminidase with an IC_50_ value of 18.0 nM [67]. Andrastone A (**251**), 16-*epi*-citreohybriddione (**252**) and citreohybriddione A (**253**) (Figure 22) were recently isolated from the marine-derived fungus *P. allii-sativi*, isolated from the deep-sea water of the western Pacific. All the isolated compounds were evaluated for their antiproliferative effects against HepG2, A549, BIU-87 (urinary bladder), BEL-7402, ECA-109 (esophageal squamous carcinoma), HelaS3 (cervix), and PANC-1 (prancreatic) human tumour cell lines; however, only **251** displayed significant activity, with selective effect against HepG2 tumour cells with an IC_50_ = 7.8 µM. Compound **251** also significantly increased caspase-3 and caspase-8 activities, but exhibited almost no effect on caspase-9. Moreover, this compound was found to increase the reporter transcriptional activation of RXRα (retinoid X receptor α) while reducing the transactivity of RXRα induced by 9-*cis*-retinoic acid in the luciferase reporter gene assay [71]. It is interesting to note that the absolute configurations of the stereogenic carbons of the sesquiterpene skeleton of **250**, i.e., C-5, C-8, C-9, C-10, C-13 and C-14 are opposite to those of **242**–**249** and **252–253**. Biogenetically, this is improbable. Even though the absolute structures of **249 [70]** and **253 [71]** were established by X-ray analysis, and those of **242**–**244** [70], **251** and **252** [71] were determined by comparison of the experimental and calculated (using TDDFT) ECD spectra, the parameters of the methods of determination of the configurations such as the flack parameter (in X-ray crystallography) and the precise wave length in the ECD curves of the experimental and calculated spectra should be duly taken into consideration.

##### Drimane Sesquiterpenes Linked to Rearranged 3,5-dimethylorsellinic Acid (Figure 23)

**Figure 23:**
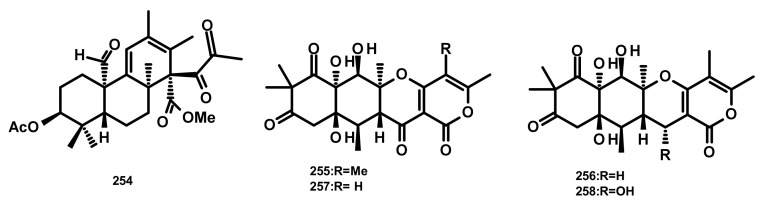
Chemical structures of **254**–**258**.

Simpterpenoid A (**254**) (Figure 23), a merosesquiterpene containing a highly functionalized cyclohexadiene with *gem*-propane-1,2-dione and methylformate groups, was isolated from the culture extract of *Penicillium simplicissimum* MA-332, obtained from the rhizospheric soil of the marine mangrove plant *Bruguiera sexangula* var. *rhynchopetala*. Compound **254** exhibited potent in vitro inhibitory activity against the influenza neuraminidase with an IC_50_ value of 8.1 nM (positive control: Oseltamivir; IC_50_ = 3.2 nM) but weak growth inhibitory activity against a plant pathogenic fungus *Physalospora piricola* [72].

##### Rearranged Drimane Sesquiterpenes Linked to an Isochromone (Figure 23)

The highly oxygenated merosesquiterpenes containing a rearranged drimane linked to an isochromone moiety, aspertetranones A–D (**255**–**258**) (Figure 23), were isolated from the culture extract of the endophytic fungus *Aspergillus* sp. ZL0-1b14 obtained from the marine green algal species of the genus *Enteromorpha*, which was collected from Jinjiang Dongshi salt pan in Fujian Province, China. Compounds **255**–**258** displayed weak inhibitory activities against TNF-α and NO production by LPS-stimulated RAW264.7 macrophages [73].

### 2.4. Meroditerpenoids

Naturally occurring meroditerpenoids can be categorized into three main classes: (i)-diterpenes combined with 3,5-dimethylorsellinic acid, (ii)-diterpenes combined with polyketides, and (iii)- diterpenes combined with indole derivatives.

#### 2.4.1. Diterpenes Linked to 3,5-Dimethylorsellinic Acid (Figure 24)

**Figure 24:**
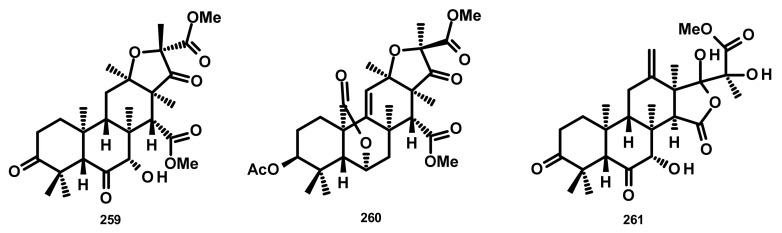
Chemical structures of **259**–**261**.

Terreusterpenes A–C (**259**–**261**) (Figure 24) were isolated from the culture extract of *Aspergillus terreus*, obtained from the inner part of the soft coral *Sarcophyton subviride* which was collected from the Xisha Island, China. Compounds **259** and **260** exhibited potent inhibitory activity against BACE1 (β-site amyloid precursor protein-cleaving enzyme 1) with IC_50_ values of 5.98 and 11.42 μM, respectively [74]. BACE1 was identified as being responsible for the formation of amyloid beta (Aβ) which is a highly aggregatory peptide segment of the membrane-associated amyloid precursor protein. Since Aβ aggregate is one of the targets for the drug discovery for Alzheimer’s disease (AD), **259** and **260** could be an interesting model for a development of AD’s drugs.

#### 2.4.2. Diterpenes Linked to Polyketides (Figure 25)

**Figure 25:**
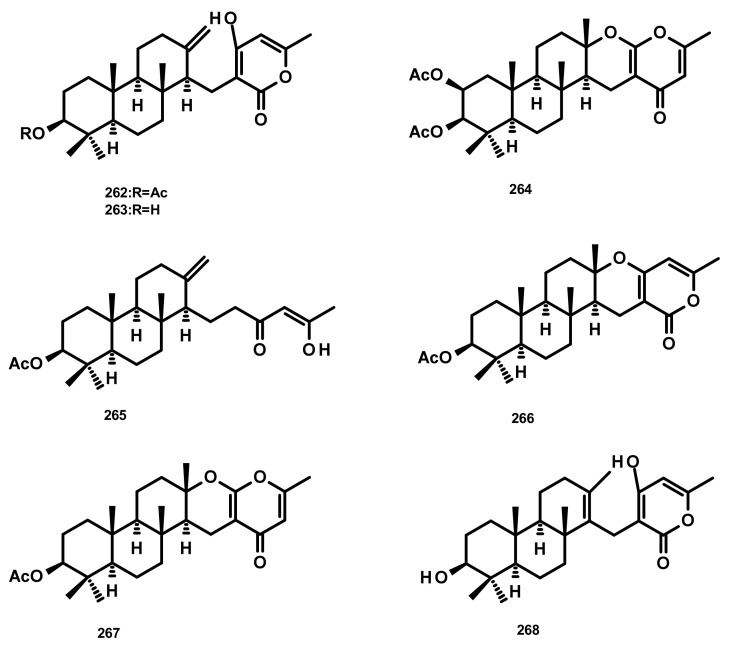
Chemical structures **262**–**268**.

Aszonapyrones A (**262**) and B (**263**) (Figure 25), two tricyclic meroditerpenes containing a 2-pyrone ring, were isolated from the culture extract of the diseased coral-derived fungus *Neosartorya laciniosa* KUFC 7896, whereas sartorypyrone B (**264**) (Figure 25) was isolated from the culture extract of the marine sponge-associated fungus *N. tsunodae* KUFC 9213 which was obtained from the marine sponge *Aka coralliphaga*, collected from the Gulf of Thailand. Compounds **262** and **264** were examined for their cytotoxic activity against MCF-7, NCI-H460 and A375-C5 (melanoma) cell lines, using the protein binding dye sulforhodamine B (SRB) method. Compound **262** displayed potent growth inhibitory activity against the three cell lines, with GI_50_ values of 13.6 ± 0.9, 11.6 ± 1.5 and 10.2 ± 1.2 μM, respectively, whereas **263** exhibited no activity at a concentration as high as 150 μM. Compound **264** also showed strong growth inhibitory activity against the same tumour cell lines, although less than that of **262**, with GI_50_ values of 17.8 ± 7.4, 20.5 ± 2.4 and 25.0 ± 4.4 μM, respectively [75]. Compound **262** also exhibited potent antibacterial activity against *S. aureus* ATCC 25,923 and *B. subtilis* ATCC 6633, with the MIC values of 8 µg/mL, and multidrug-resistant *S. aureus* MRSA and *Enterococcus* spp. VRE isolates, with the MIC values of 8 and 16 µg/mL, respectively. Although **262** showed partial synergism with the antibiotics oxacillin and ampicillin against MRSA and VRE isolates, respectively, it showed a clear synergistic effect with vancomycin (VA) against the two VRE isolates tested (*E. faecalis* W1 and *E. faecium* W5). Moreover, **262**, at the MIC and 2 MIC concentrations, completely inhibited biofilm formation of *S. aureus* ATCC 25,923, *B. subtilis* ATCC 6633 and the multidrug-resistant *S. aureus* B1 and *E. faecalis* W1. However, *S. aureus* ATCC 25,923 and *S. aureus* B1 produced more biofilm at the subinhibitory concentration (1/2 MIC) of **262** [76]. A new aszonapyrone analogue, sartorypyrone C (**268**) (Figure 25), was isolated from the culture extract of the marine-derived fungus *N. paulistensis* KUFC 7897, obtained from the marine sponge *Chondrilla australiensis*, collected from the Gulf of Thailand [76]. Sartorenol (**265**) (Figure 25), a triclyclic meroditerpene, was isolated, together with **262** and chevalone B (**266**) (Figure 25), from the algicolous fungus *N. takakii* KUFC 7898, obtained from the marine macroalga *Amphiroa* sp., collected in the Gulf of Thailand. Compound **265** showed no antibacterial activity against the above-mentioned four reference strains and environmental multidrug-resistant isolates [77]. Chemical examination of the marine-derived fungus *N. siamensis*, isolated from the sea fan *Rumphella* sp. which was collected from the Andaman Sea of Thailand, led to the isolation of chevalone C (**267**) (Figure 25). Compound **267** exhibited moderate cytotoxicity against three tumour cell lines including colon HCT-116, liver HepG2 and melanoma A375 with IC_50_ values ranging from 24 to 153 μM [78]. Compound **266** was also recently reported from *Aspergillus* sp. H30 which was isolated from a sea cucumber *Cucumaria japonica*, collected from the South China Sea. Although **266** displayed weak antimicrobial activity against *C. albicans* SC5314 and methicillin-resistant *S. aureus* (MRSA), it exhibited cytotoxic activity against BC1 (lymphoma), KB, and NCI-H187 tumour cell lines [79].

#### 2.4.3. Indole Diterpenoids (Figure 26–28)

**Figure 26:**
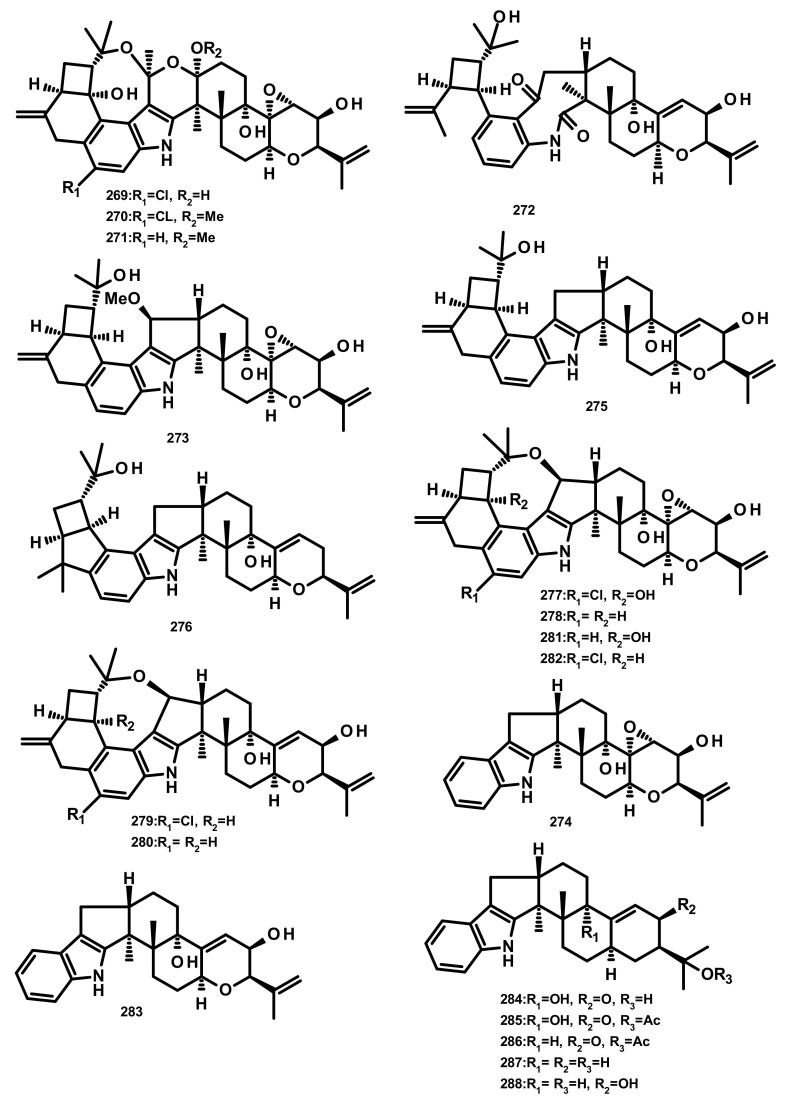
Chemical structures of **269**–**288**.

Rhizovarins A–F (**269**–**274**), secopenitrem D (**275**), PC-M4 (**276**), penitrems A–F (**277**–**282**), penijanthine A (**283**), paxilline (**284**), 1′-*O*-acetylpaxilline (**285**), 4b-deoxy-1′-*O*-acetylpaxilline (**286**), 3-deoxo-4b-deoxypaxilline (**287**) and 3b-hydroxy-4b-desoxypaxilline (**288**) (Figure 26) are indoloditerpenoids isolated from the culture extract of the endophytic fungus *Mucor irregularis* QEN-189 which was obtained from the fresh inner tissue of the stem of the mangrove plant *Rhizophora stylosa*, collected on Hainan Island. Compounds **269**, **270**, **277**, **282** and **288** exhibited growth inhibitory activity against human A-549 and HL-60 cancer cell lines (IC_50_ values ranging from 2.6–11.5 μM) whereas **281** was active only against A-549 cancer cell line [80].

**Figure 27:**
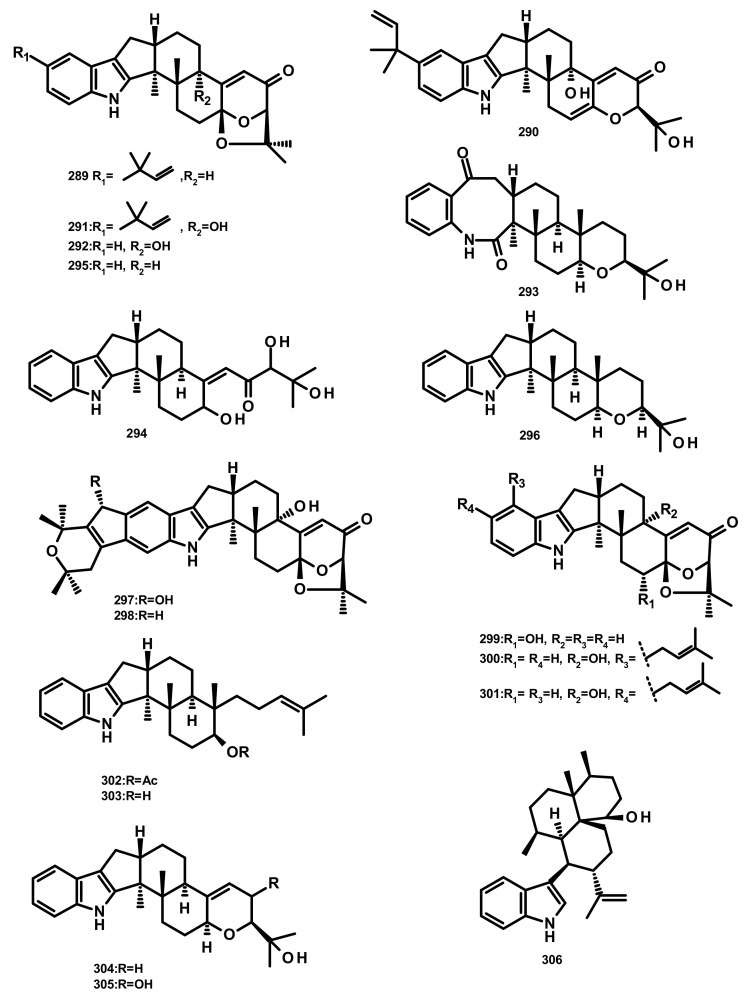
Chemical structures **289**–**306**.

Four indoloditerpenes, including (2*R*,4b*R*,6a*S*,12b*S*,12c*S*,14a*S*)-4b-deoxy-β-aflatrem (**289**), (2*R*,4b*S*,6a*S*,12b*S*,12c*R*)-9-isopentenylpaxilline D (**290**), *β*-aflatrem (**291**) and paspalinine (**292**) (Figure 27) were reported from the culture extract of *Aspergillus flavus* OUCMDZ-2205 isolated from the marine prawn (*Penaeus vannamei*). Compound **289** displayed weak antibacterial activity against *S. aureus* with a MIC value of 20.5 μM. Additionally, **289** and **290** were able to arrest the A549 cell cycle in the *S* phase at a concentration of 10 μM. Moreover, **289** displayed PKC-beta inhibition with an IC_50_ value of 15.6 μM [81]. Two indoloditerpenes **293** and **294** (Figure 27) were isolated, together with **292,** paspalicine (**295**) and paspaline (**296**) (Figure 27), from two marine-derived *Aspergillus* sp. AF-119 and *Aspergillus* sp. JQG 1-6f. Compounds **289** and **290** displayed significant antibacterial activity against a panel of bacterial isolates including *S. aureus*, *B. subtilis* and *E. coli* but are void of antifungal activity [82]. The culture extract of the sea anemone-derived fungus *Penicillium* sp. furnished **296**, 22-hydroxylshearinine F (**297**), shearinine F (**298**), 6-hydroxylpaspalinine (**299**), paspalitrem C (**300**), paspalitrem A (**301**), 7-*O*-acetylemindole SB (**302**), emindole SB (**303**), 3-deoxo-4b-deoxypaxilline (**304**), PC-M6 (**305**) and 10, 23-dihydro-24, 25-dehydroaflavinine (**306**) (Figure 27). Compounds **297**–**306** were tested for their antibacterial activity against several human-, aqua-, and plant-pathogenic microbes; however, the tested compounds displayed antimicrobial activity in micromolar range against *P. aeruginosa*, *E. coli, Vibrio parahaemolyticus* and *V. alginolyticus* [83].

**Figure 28:**
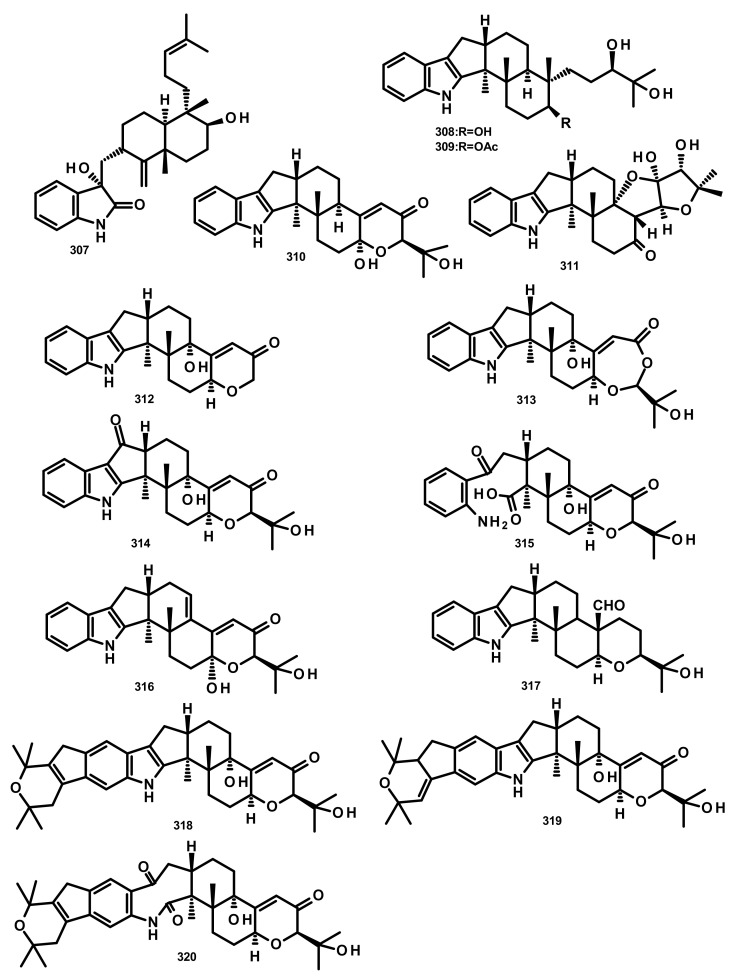
Chemical structures of **307**–**320**.

Penicindopene A (**307**) (Figure 28), an indole-bicylic diterpene, was isolated from the culture extract of *Penicillium* sp. YPCMAC1, obtained from the deep-sea water at a depth of 4500 m of the Yap Trench in the West Pacific Ocean. Compound **307** displayed a moderate antitumour activity against A549 and HeLa cell lines with IC_50_ values of 15.2 and 20.5 µM, respectively [84]. Two indole-tricyclic diterpenes, penijanthines C (**308**) and D (**309**) (Figure 28), were reported together with **305** and 7-hydroxy-13-dehydroxypaxilline (**310**) (Figure 28), from the marine-derived fungus *Penicillium janthinellum*, which was isolated from a marine sediment collected from the Bohai Sea. Compounds **305**, **308**, **309** and **310** displayed significant growth inhibitory activity against Gram-negative halophilic pathogenic bacteria *V. anguillarum, V. parahemolyticus*, and *V. alginolyticus* with MIC values ranging from 3.1 to 50.0 µM [85] (Figure 28).

Previously unreported penerpenes E–I (**311**–**315**) (Figure 28), along with the known congeners including **293**, **304**, 7-hydroxypaxilline-13-ene (**316**), paspaline B (**317**), pyrapaxilline (**318**), shearinine B (**319**) and shearinine P (**320**) (Figure 28), were isolated from the marine-derived fungus *Penicillium* sp. KFD28 which was obtained from a bivalve mollusc, *Meretrix lusoria*, collected from Haikou Bay. Compounds **311**, **312**, **314** and **316** displayed moderate protein tyrosine phosphatase 1B (PTP1B) inhibitory activity with IC_50_ values of 14, 27, 23, and 13 μM, respectively [86].

## 3. Conclusions and Prospects

The marine world represents the largest and most diverse ecosystem on earth. Since 1950′s, marine natural products chemists have raised the prospects of marine natural products (MNPs) as a great potential and renewable pipelines for compounds of a huge interest in pharmaceutical, nutraceutical and cosmetic industries. Marine microorganisms have become increasingly attractive as sources of compounds with unique structural features and unprecedented pharmacological activities. Marine-derived fungi represent an important source of MNPs due to their variable habitats from the tropics to the polar regions, from the surface to the seafloor and even at such extreme temperature and pressure as in a hydrothermal vent. Moreover, marine-derived fungi are also a prolific source of secondary metabolites capable of synthesizing a myriad of chemical classes of compounds. One of the most interesting classes of fungal secondary metabolites is meroterpenoids. According to our literature search over the period of January 2009 to the end of December 2019, 320 marine meroterpenoids have been reported from a myriad of marine-derived fungi from different habitats, many of which possess unique structural features and undescribed biological and pharmacological activities. At present, natural products from marine-derived fungi have not yet attained the status of the compounds produced by other marine organisms in the pharmaceutical industry. However, this is a question of time since many compounds produced by terrestrial fungi have been approved and successfully marketed as antibiotics, anticholesterolemic, among others. Moreover, many compounds have been successfully explored as cosmeceuticals and nutricosmetics whose market is in a marked expansion. Thus, the contribution of MNPs is undoubtedly vital not only for the pharmaceutical industry but also for other health industries, as well as for the preservation of the marine environment. Marine fungi are undoubtedly an important reservoir of a hidden treasure awaiting to be explored. With a rapid advancement of culture techniques, genome mining to uncover biosynthetic gene clusters, extraction processes and molecular techniques for bioassays, marine-derived fungi could become a great potential to provide valuable compounds as leads for drug development to combat many diseases, to maintain our healthy appearance and even for molecular tools to unlock the mechanisms of many rare and incurable diseases. With modern biotechnological processes, marine-derived fungi can be a huge renewable and untapped source of bioactive natural products while keeping the marine environment intact.
